# Supplementary material for: Resting state functional connectivity patterns associate with alcohol use disorder characteristics: Insights from the triple network model
Source: Neuroimage Clin. 2025 Dec 31;49:103939. doi: 10.1016/j.nicl.2025.103939 (PMC12811491; doi:10.1016/j.nicl.2025.103939)
Supplement: Supplementary Data 1 [file mmc1.docx]

Supplementary Material


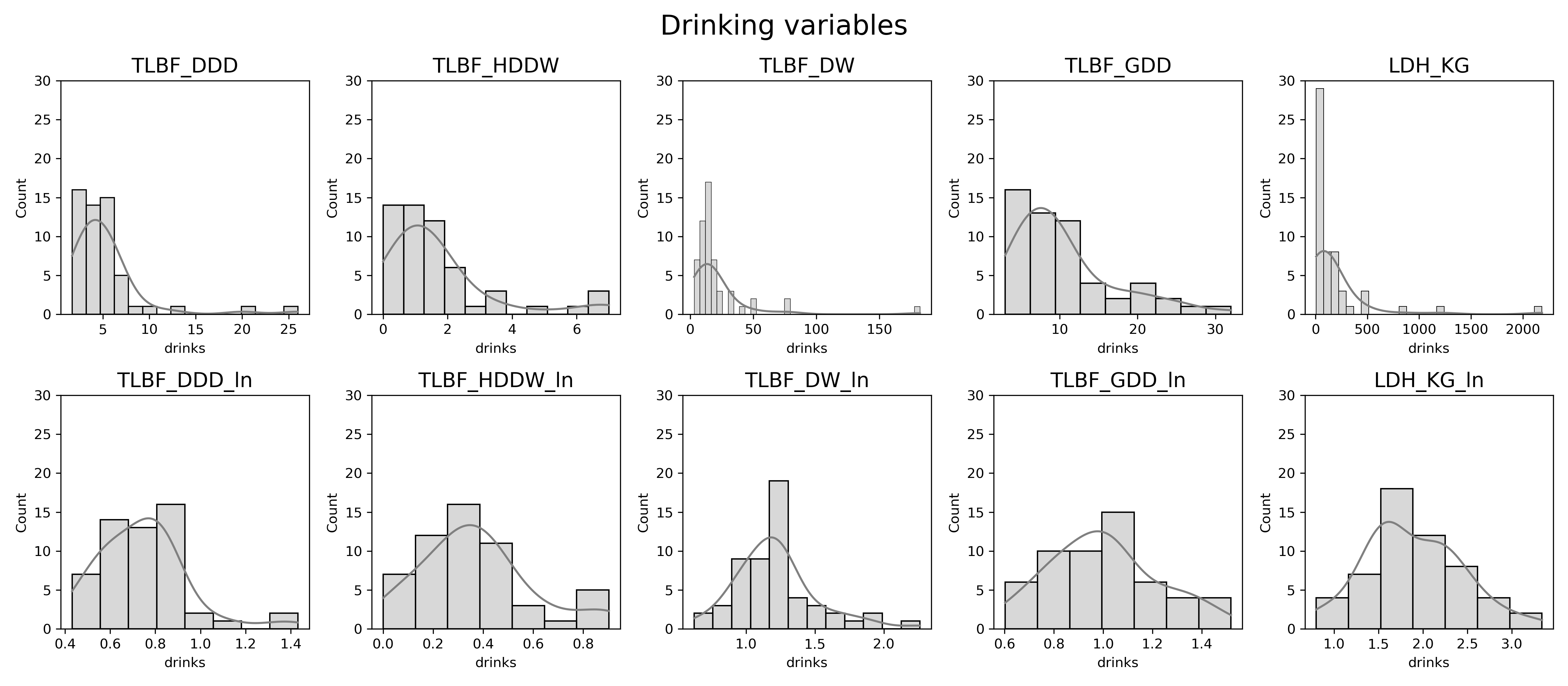
**Figure S1.** Drinking related variables capture recent and long-term drinking patterns. TLFB_DDD: drinks per drinking day, TLFB_HDDW: heavy drinking days per drinking week, TLFB_DW: drinks per week, TLFB_GDD: maximum drinks per drinking day, LDH_KG: lifetime drinking history in kilograms. The original variables presented skewed distributions, so they were logarithmically transformed and used as input to PCA to compute a composite Drinking variable.


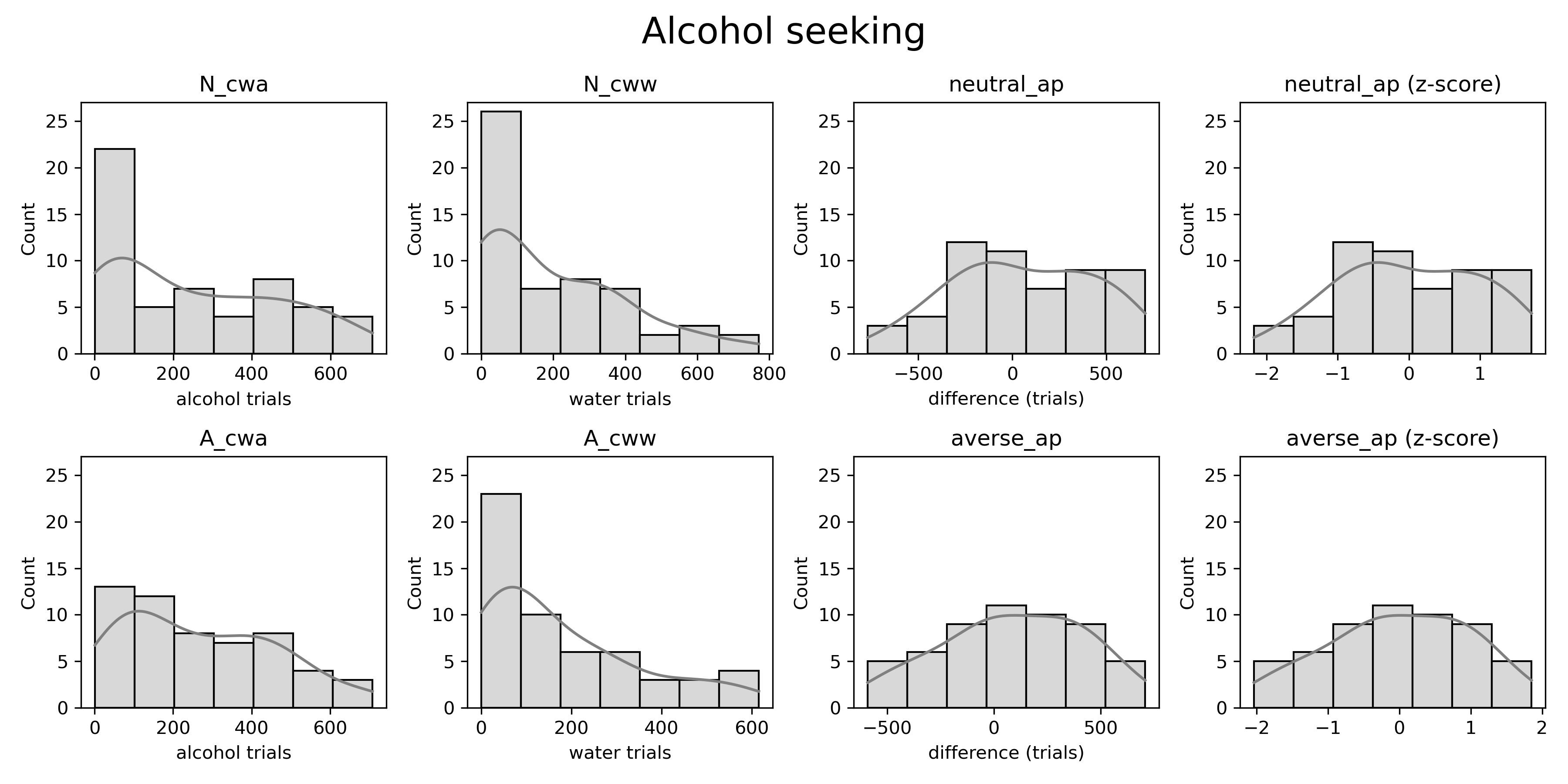
**Figure S2.** Cumulative work (number of trials) for alcohol and water during the alcohol self-administration sessions. A_cwa: cumulative work for alcohol in aversive session. A_cww: cumulative work for water in aversive session. N_cwa: cumulative work for alcohol in neutral session. N_cww: cumulative work for water in neutral session.


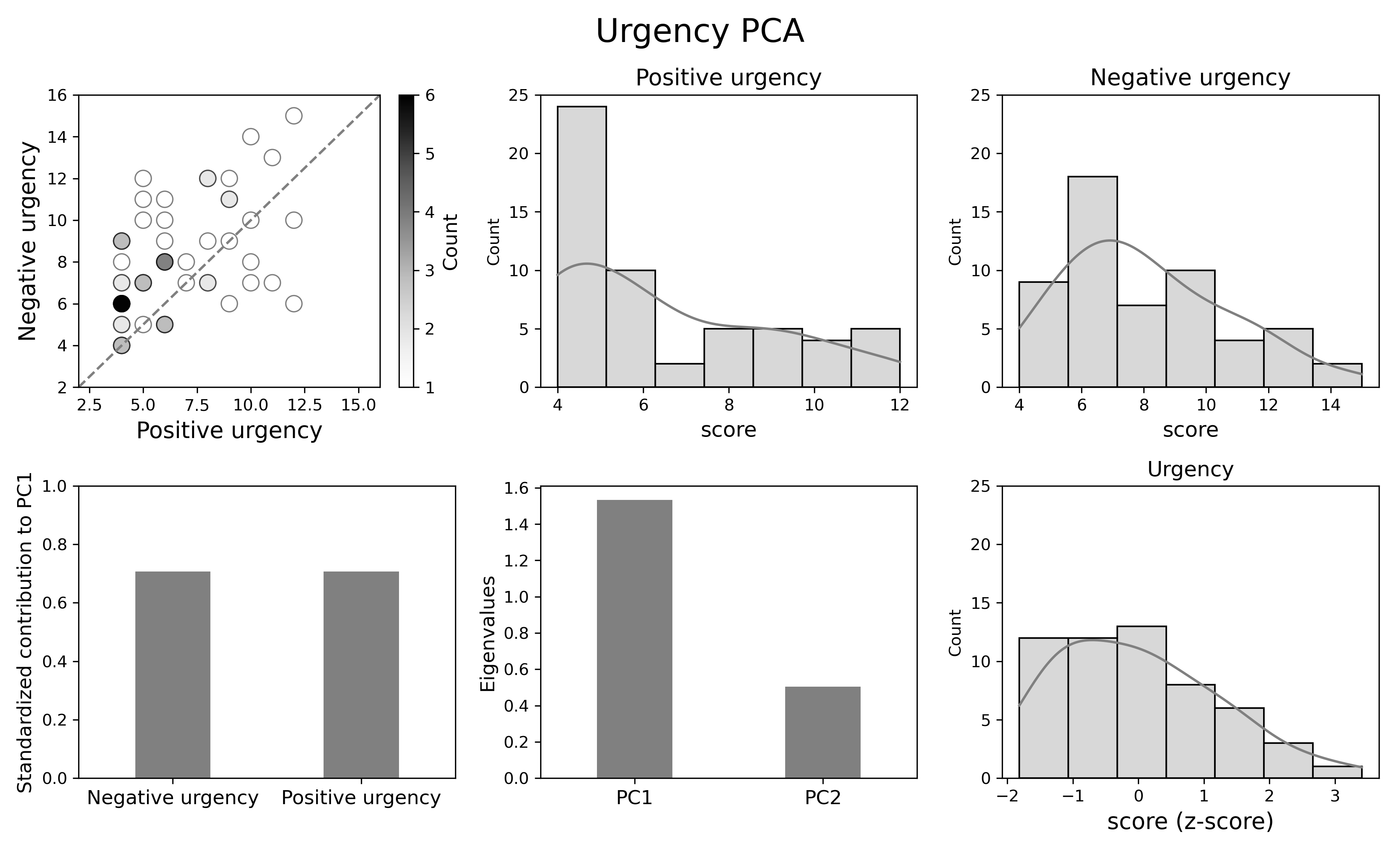
**Figure S3.** Positive and negative urgency are combined into a single urgency variable using PCA (first principal component explained 75% of the variance). The contributions of each individual urgency variable to the first PCA are similar.


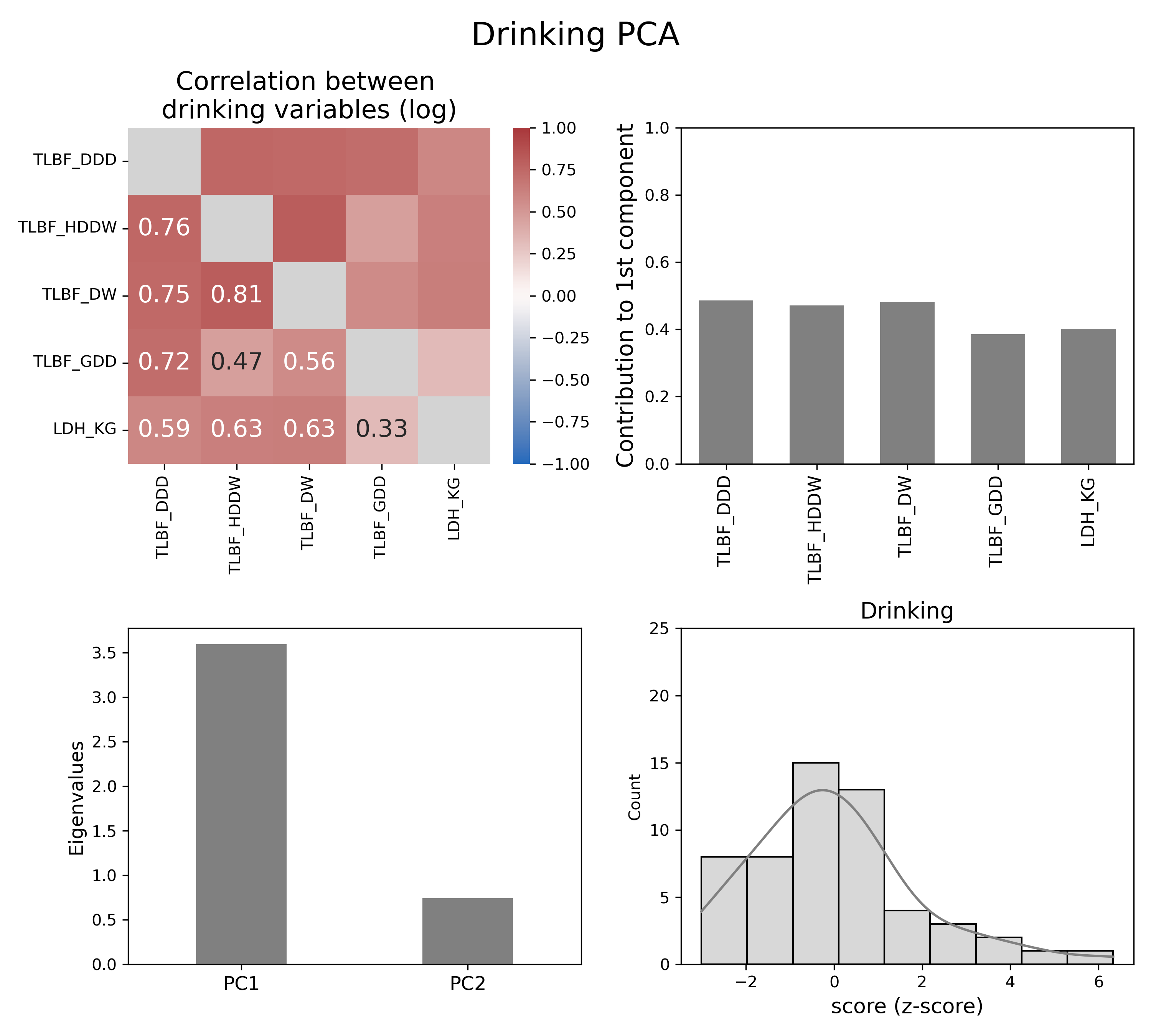
**Figure S4.** Drinking variables assessing recent (TLFB) and lifetime drinking (LDH**)** were combined into a single composite drinking variable to which all variables contributed similarly. The first principal component PCA explained 71% of the variance.


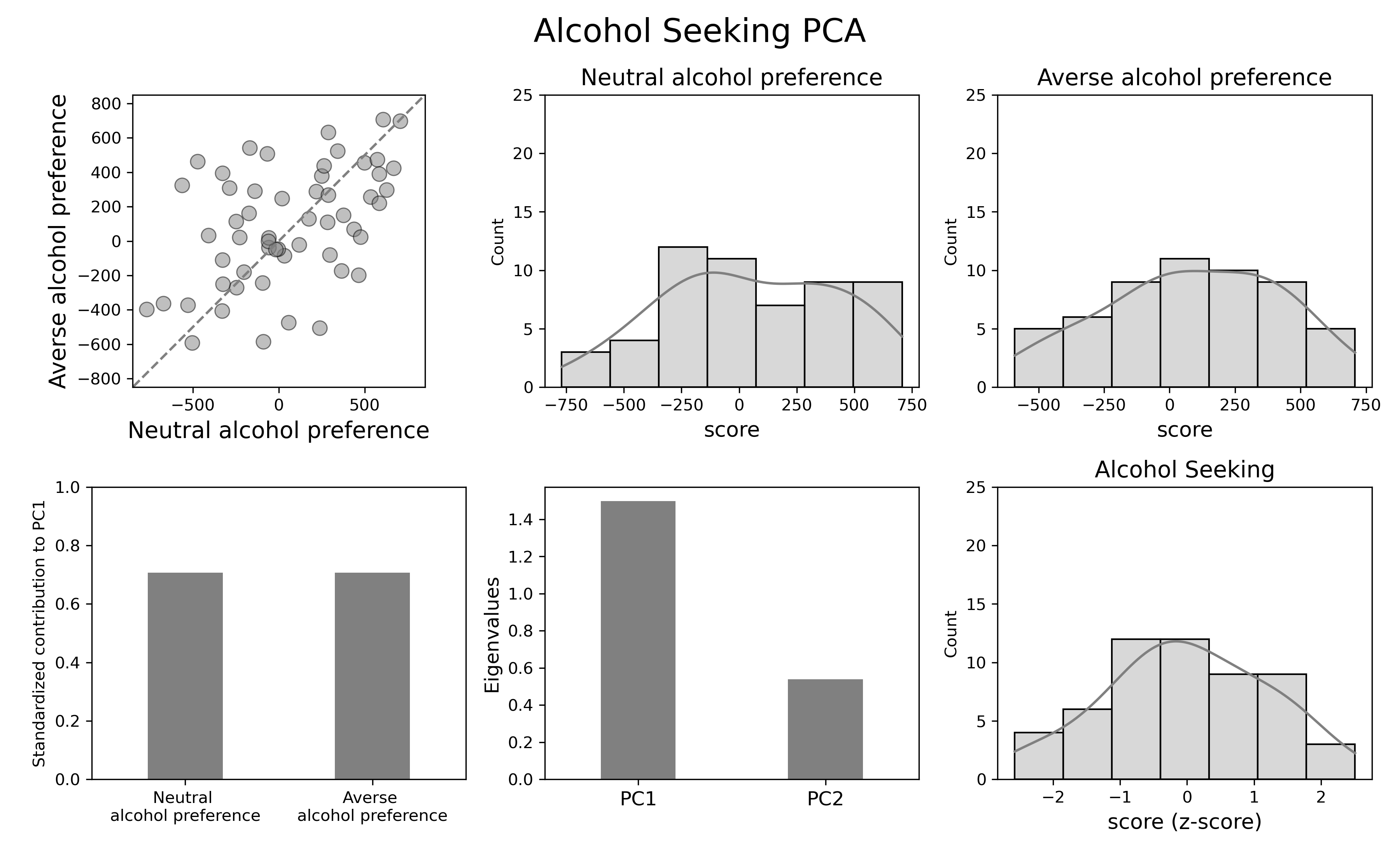
**Figure S5.** Neutral and averse alcohol preferences were combined into a single alcohol seeking variable comprising the general willingness to work for alcohol across both neutral and aversive sessions (first principal component explained 74% of the variance). Contributions of each individual variable to the first PCA are similar.


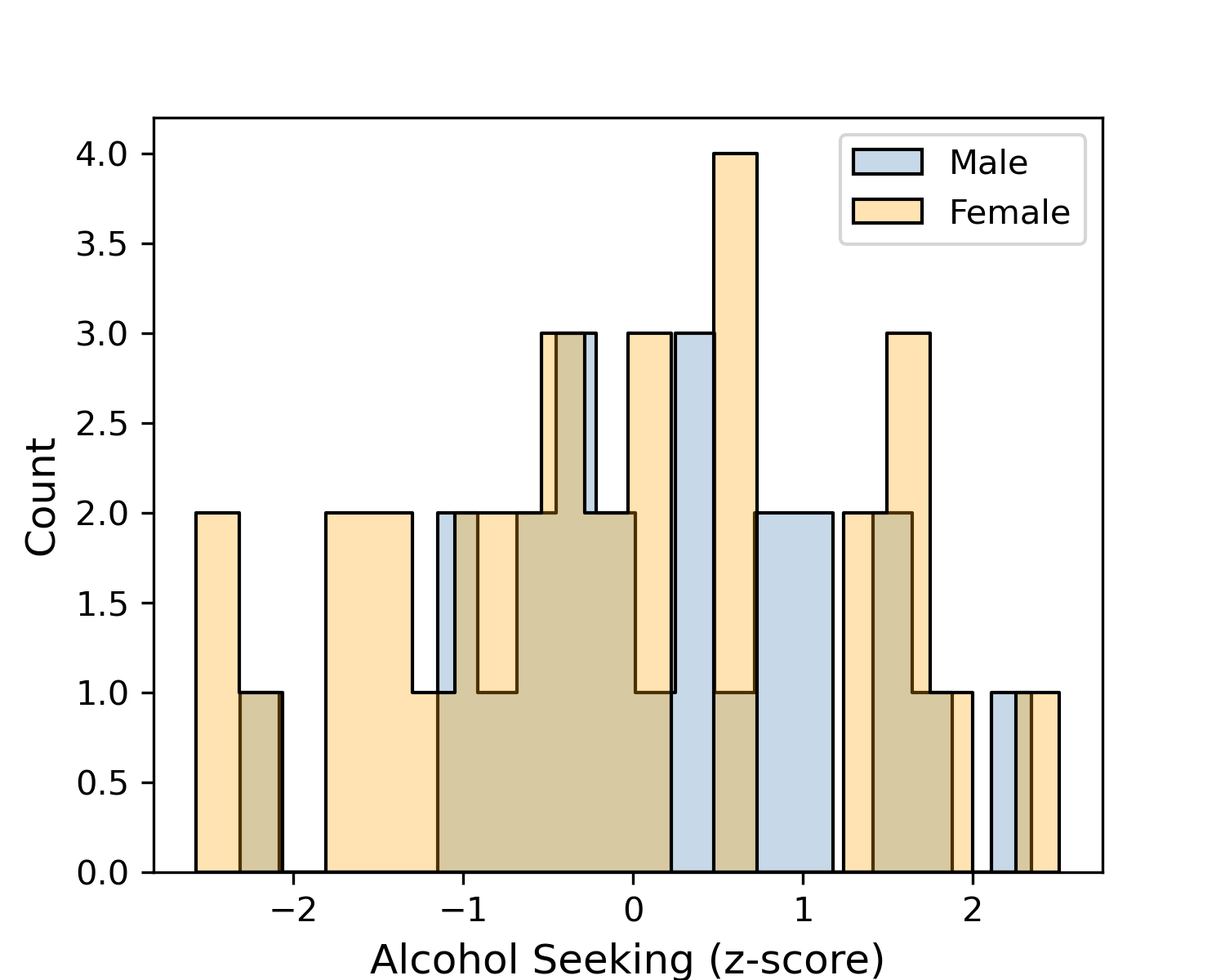


**Figure S6**. Alcohol seeking distributions for male and female participants. There were no significant differences by sex (unpaired t-test, t=1.10 p=0.28).


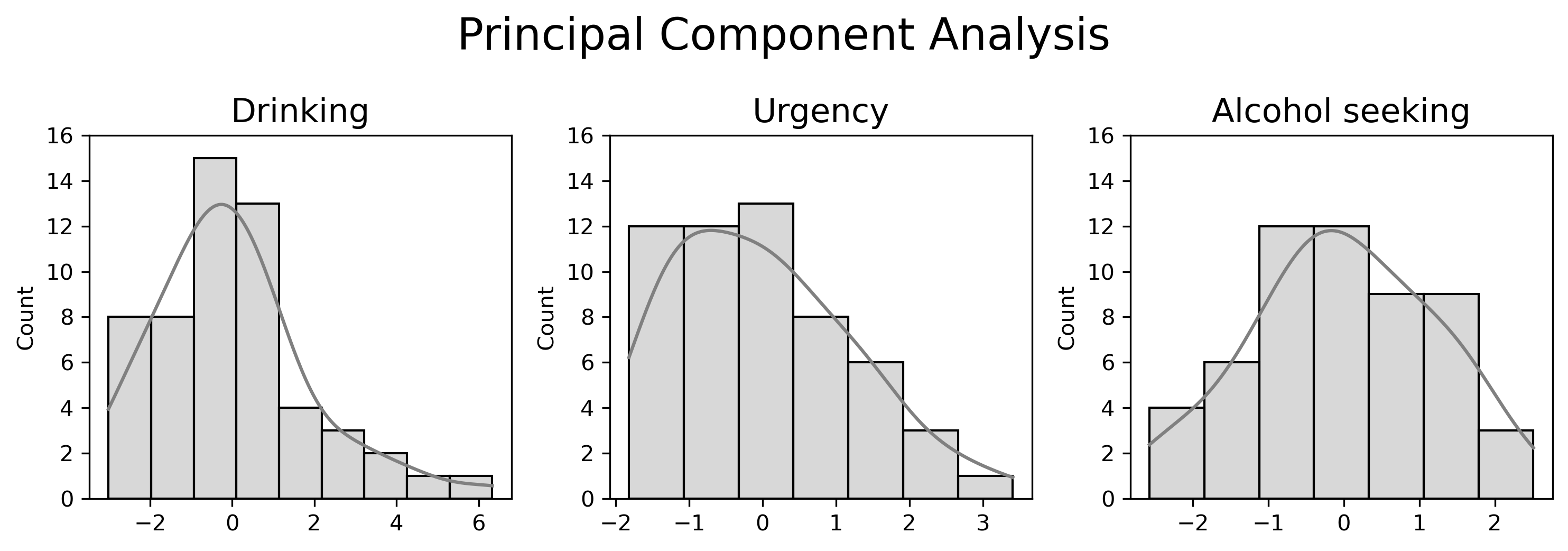
**Figure S7.** The first principal component was obtained for each of the following three sets of variables. **Drinking:** 1^st^ component of TLFB_DDD, TLFB_HDDW, TLFB_DW, TLFB_GDD, LDH_KG (71% explained variance). **Urgency:** 1^st^ component of positive and negative urgency (75% explained variance). **Alcohol seeking:** 1^st^ component of aversive and neutral alcohol preference (74% explained variance).


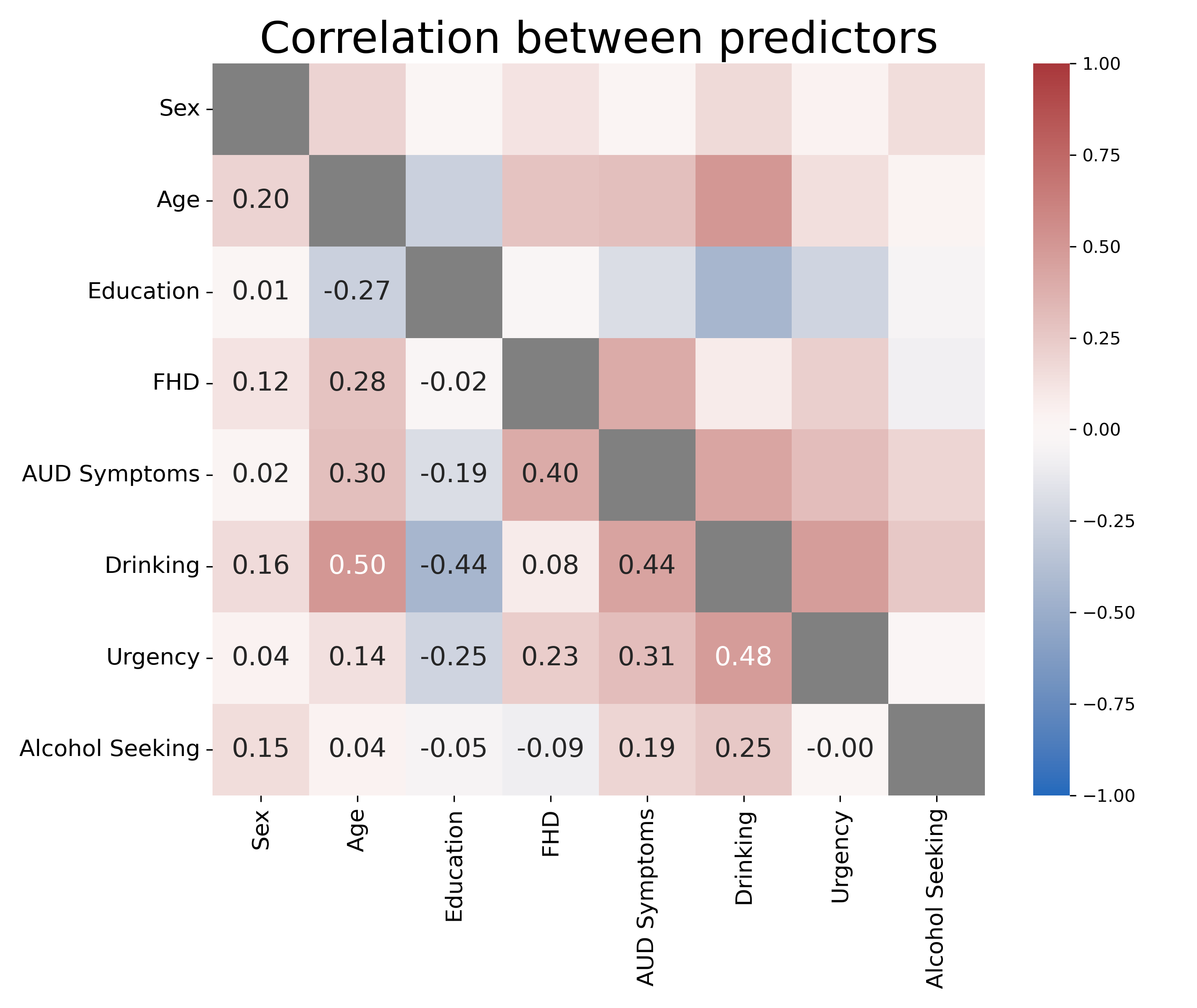
**Figure S8.** Pairwise Pearson’s correlation coefficients between the variables spanning the phenotype domain used as input to the Partial Least Square model.


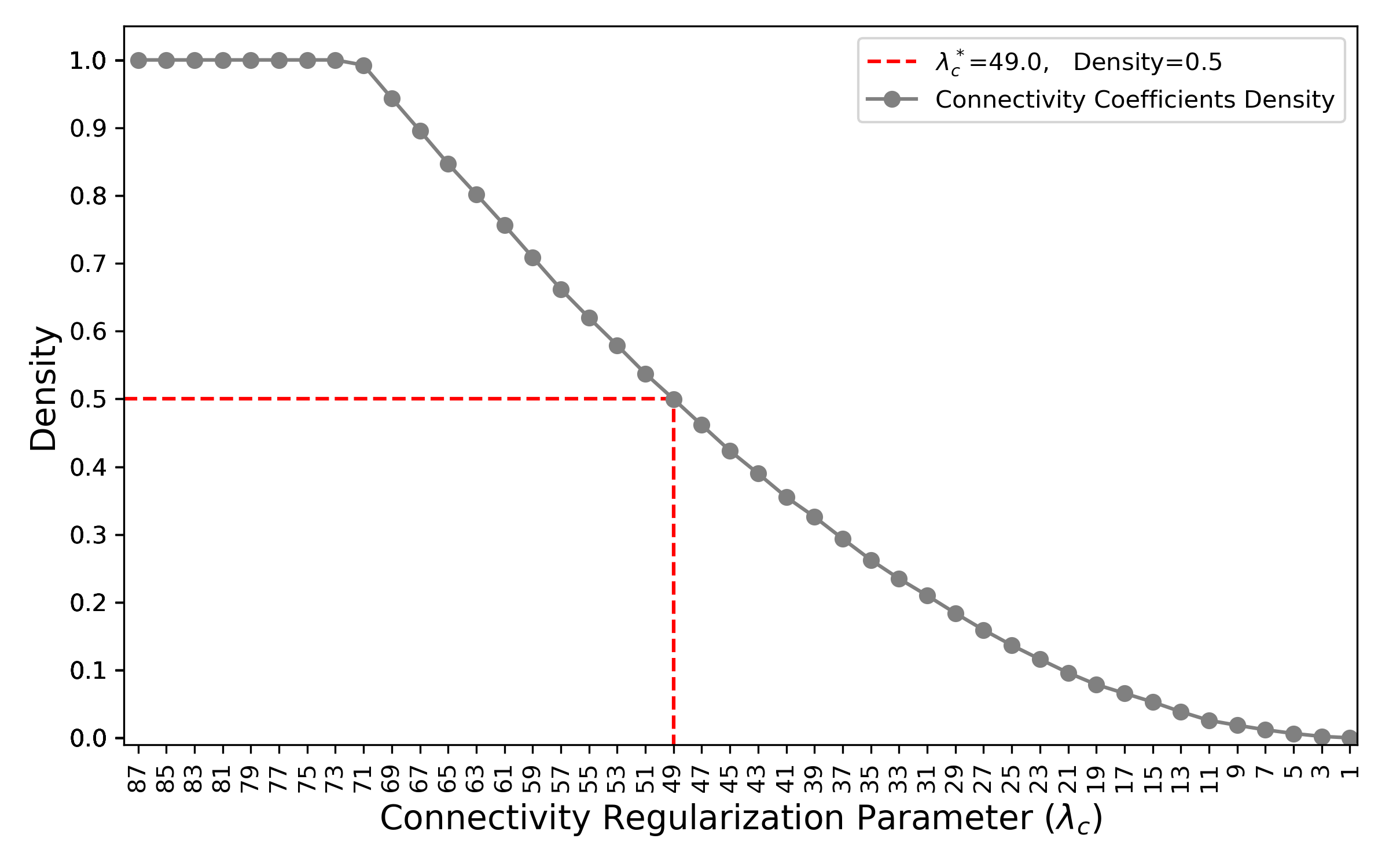
**Figure S9.** The Partial Least Squares regularization parameter (lambda) was set to the value 49.0. At this value, the density of the connectivity solution (number of functional edges participating in the solution) is 50%.


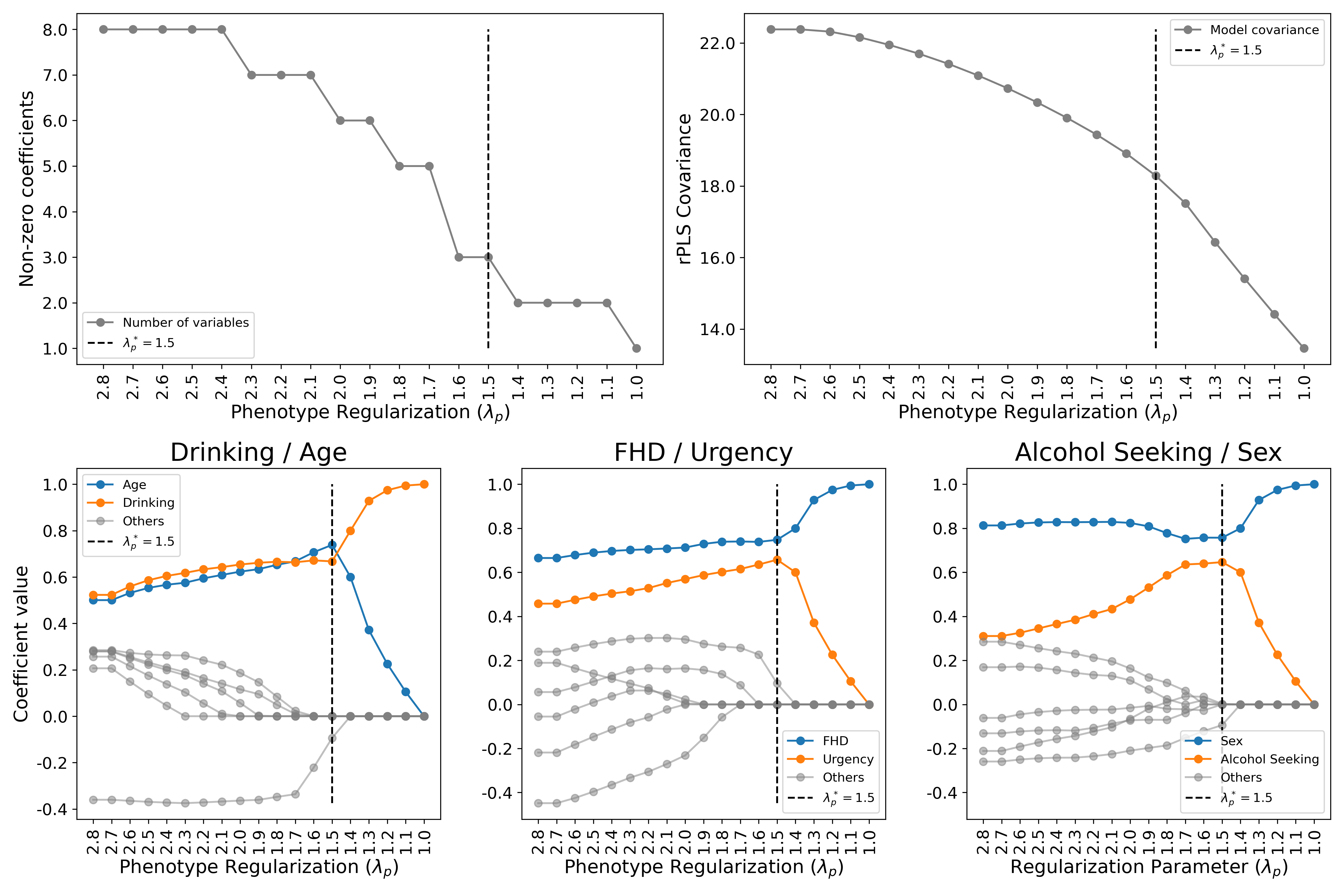
**Figure S10**. The regularization parameter for the phenotypic domain (1.5 indicated by the gray horizontal line) was selected based on the number of non-zero coefficients in component 1.


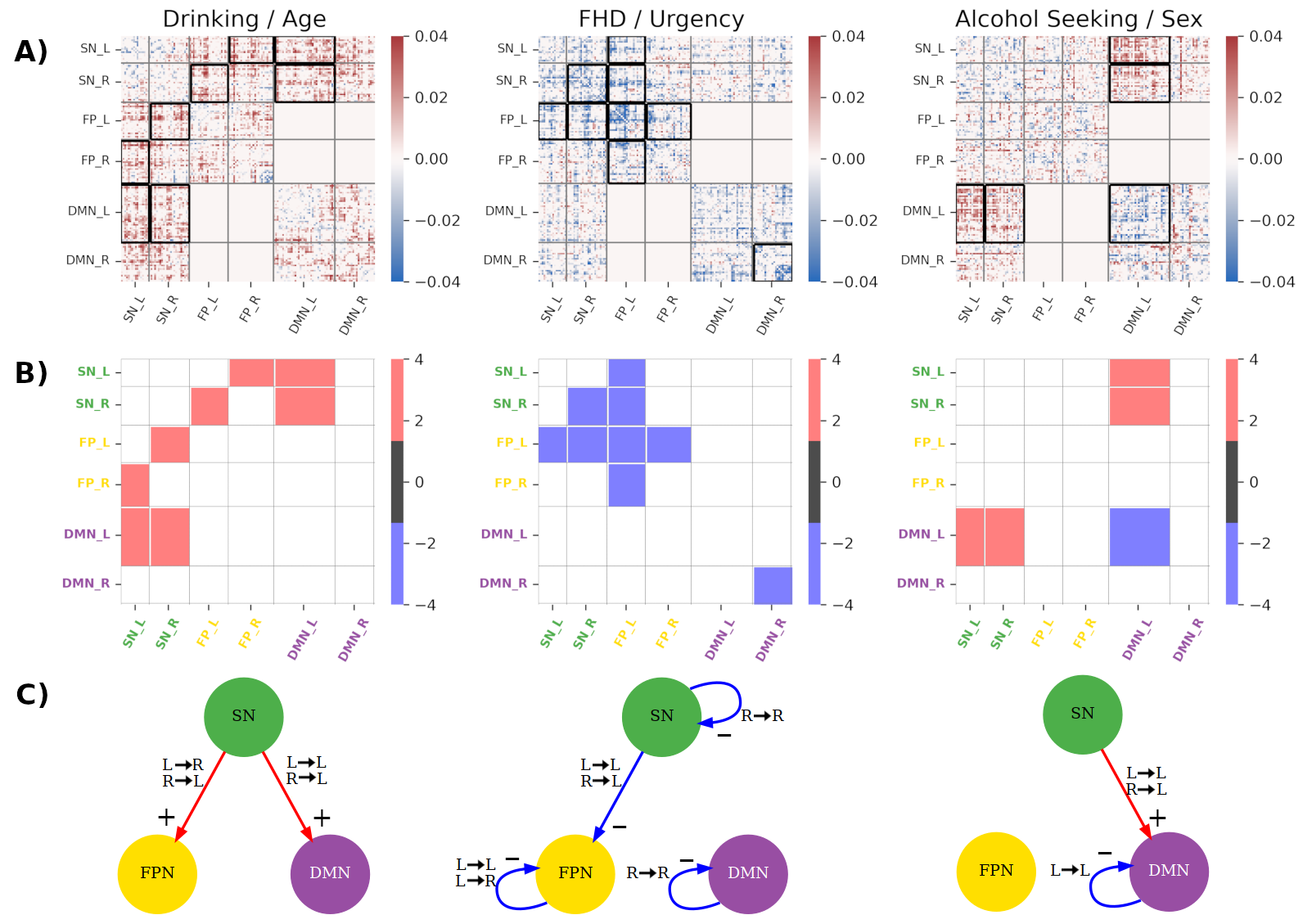
**Figure S11.** Statistical significance of within and between lateralized network interactions for each rPLS component. The statistical significance of the interactions was assessed using a null model distribution for interaction strength. Significant network interactions are shown as block contours (A), and the sign of the interaction (B) is denoted by red (positive) and blue (negative). Resulting diagrams incorporating directionality from the TNM are shown in (C).


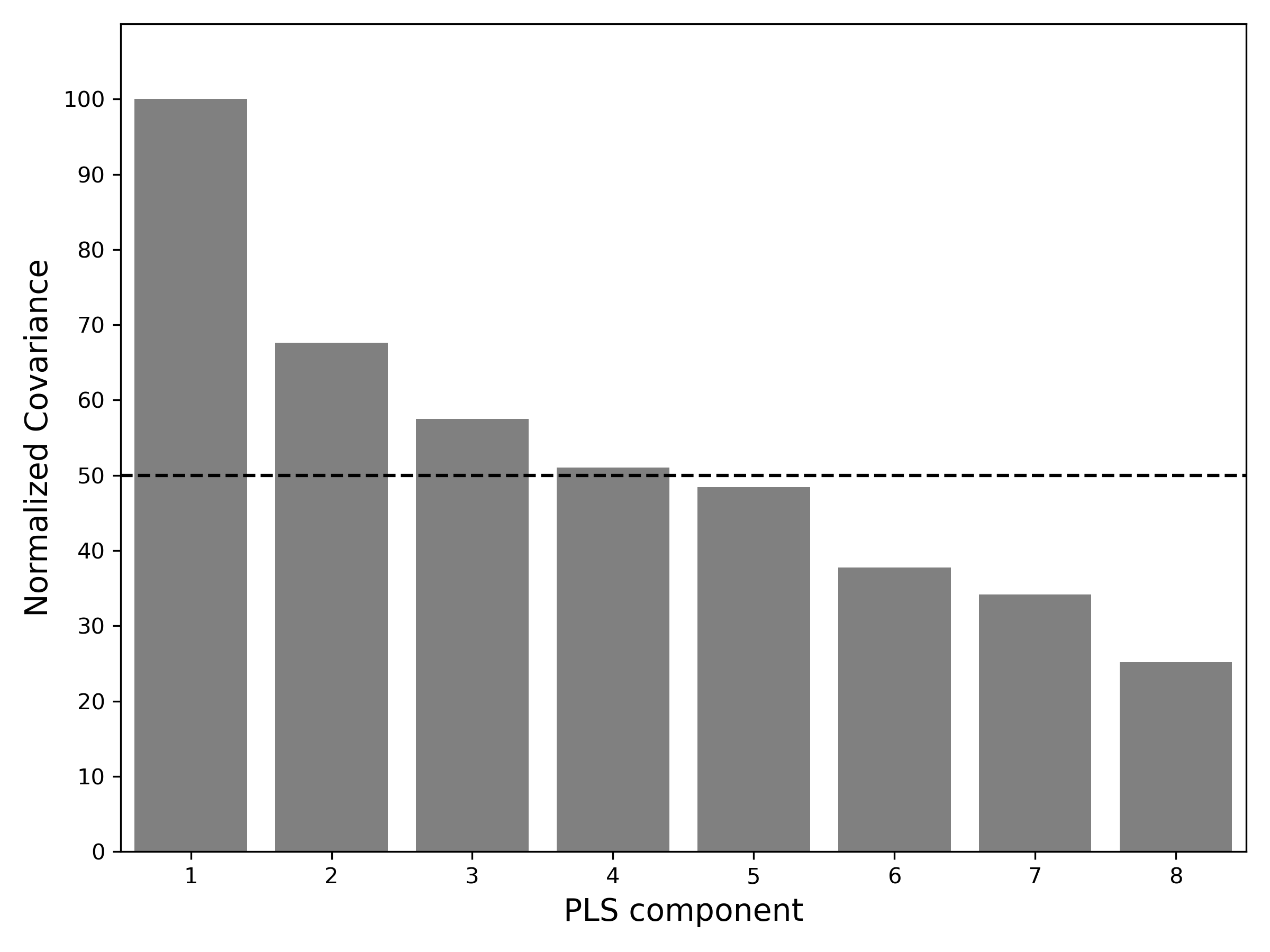
**Figure S12**. Covariance associated with each of the eight PLS components, normalized with respect to the maximum covariance (Component 1: 100%). A threshold criterion of 50% for the relative covariance retained the first four PLS components for subsequent analyses.


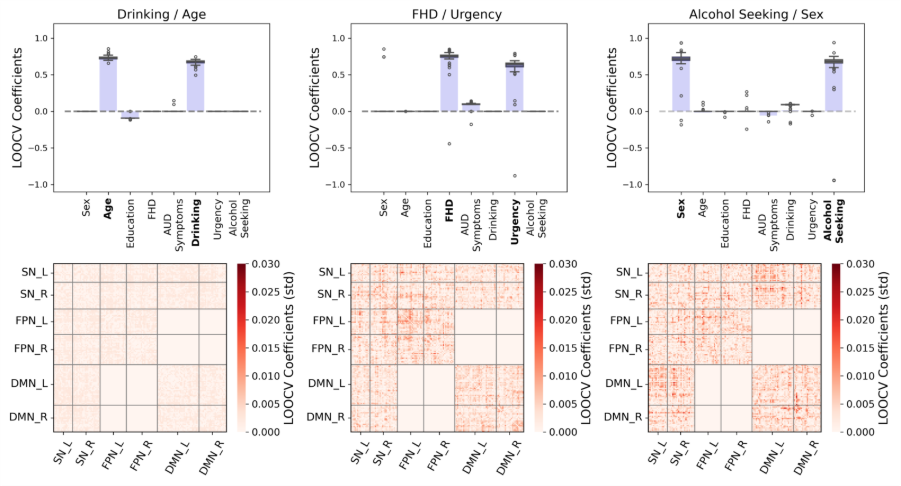
**Figure S13.** Leave-one-out cross validation of rPLS results (LOOCV; N=55) for the regularized PLS components. Top row: variability of the phenotypic characteristic coefficients across the LOOCV runs (boxplots) and full cohort coefficients for reference (purple bars). Bottom row: variability (standard deviation) of coefficients associated with each functional coupling along the LOOCV runs for each component. Interactions between FPN and DMN are excluded from the analysis following the assumptions of the TNM.


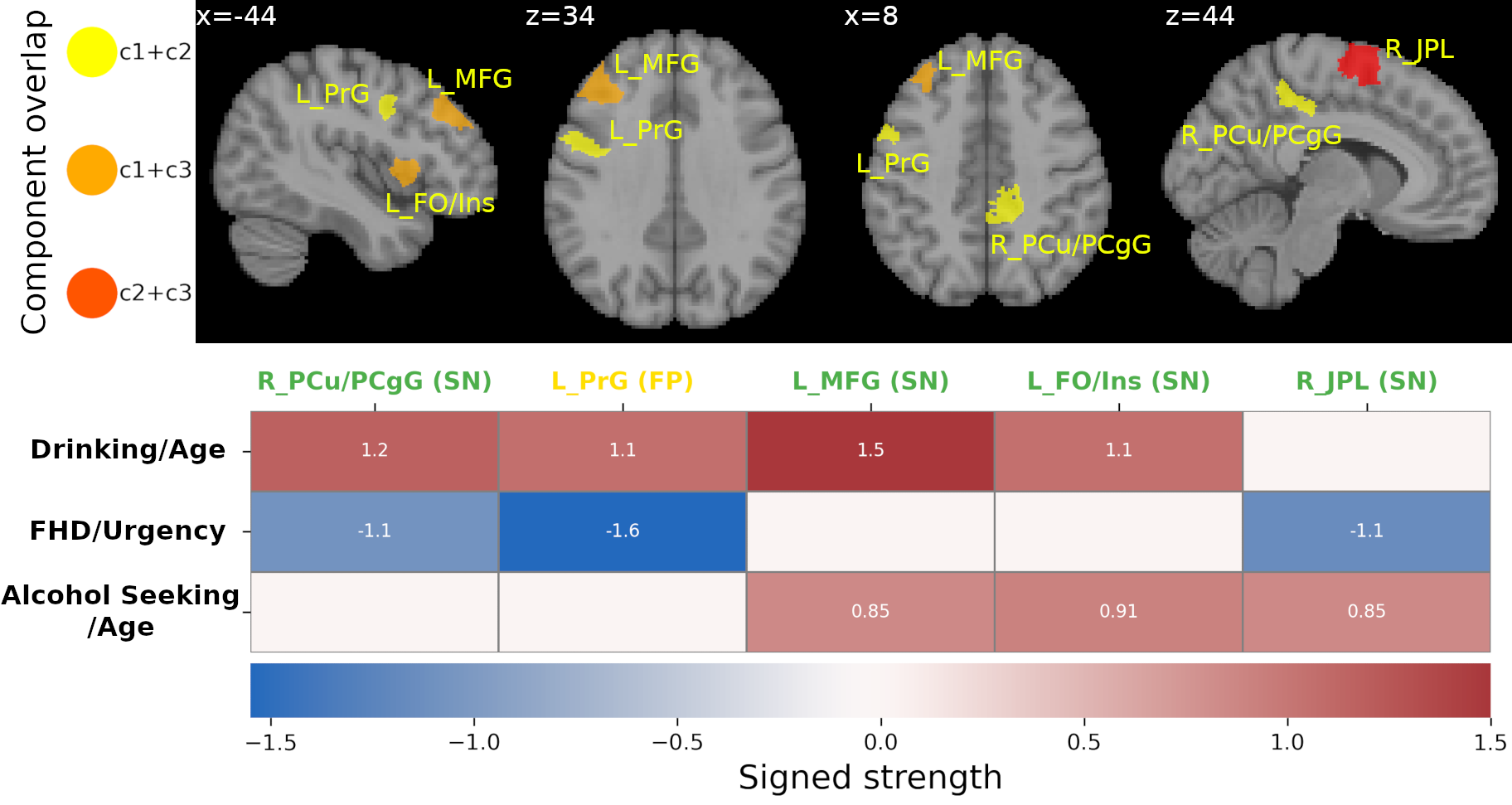
**Figure S14.** Overlapping regions between rPLS components. Among the top contributing regions in each alcohol-related component, five regions were found to participate in more than one component: the right precuneus/posterior cingulate gyrus (PCu/PCgG), left precentral gyrus (PrG), left middle frontal gyrus (MFG), left frontal operculum/insula (FO/Ins), and right juxtapositional lobule cortex (JPL). SN: Salience Network, FP: Frontoparietal Network.

|  | Drinking/Age Component | | | | | | |
| --- | --- | --- | --- | --- | --- | --- | --- |
|  | Network | Hemisphere | Schaefer 300 index | Schaefer 300 region | Description | Sign | Strength |
| 1 | SN | R | 214 | SalVentAttnA_ParOper_4 | SMG | + | 1.73 |
| 2 | FPN | R | 248 | ContA_IPS_3 | SPL/SMG | + | 1.58 |
| 3 | SN | R | 229 | SalVentAttnB_PFCl_2 | FP | + | 1.54 |
| 4 | SN | R | 212 | SalVentAttnA_ParOper_2 | PO | + | 1.52 |
| 5 | SN | L | 76 | SalVentAttnB_PFCl_2 | MFG | + | 1.50 |
| 6 | SN | L | 73 | SalVentAttnA_FrMed_2 | SFG | + | 1.29 |
| 7 | SN | R | 217 | SalVentAttnA_Ins_2 | pINS | + | 1.27 |
| 8 | SN | L | 68 | SalVentAttnA_Ins_3 | CO/mINS | + | 1.25 |
| 9 | SN | L | 72 | SalVentAttnA_FrMed_1 | ACgG/JPL | + | 1.20 |
| 10 | SN | R | 222 | SalVentAttnA_ParMed_2 | PCu/PCgG | + | 1.17 |
| 11 | DMN | L | 113 | DefaultA_PFCd_2 | SFG/MFG | + | 1.13 |
| 12 | SN | L | 75 | SalVentAttnB_PFCl_1 | FP | + | 1.10 |
| 13 | FPN | L | 99 | ContA_PFCl_4 | PrG | + | 1.06 |
| 14 | DMN | R | 284 | DefaultB_AntTemp_1 | R-TP | + | 1.05 |
| 15 | SN | L | 75 | SalVentAttnB_Ins_2 | FO/Ins | + | 1.05 |

|  | Family History/Urgency Component | | | | | | |
| --- | --- | --- | --- | --- | --- | --- | --- |
|  | Network | Hemisphere | Schaefer 300 index | Schaefer 300 region | Description | Sign | Strength |
| 1 | SN | R | 223 | SalVentAttnA_ParMed_3 | PoG/PrG | - | 1.59 |
| 2 | FPN | L | 99 | ContA_PFCl_4 | PrG | - | 1.55 |
| 3 | SN | R | 215 | SalVentAttnA_PrC_1 | PrG | - | 1.31 |
| 4 | FPN | L | 100 | ContA_Cingm_1 | ACgG | - | 1.30 |
| 5 | SN | L | 71 | SalVentAttnA_ParMed_2 | PCu | - | 1.28 |
| 6 | DMN | R | 288 | DefaultB_PFCv_2 | IFG | - | 1.23 |
| 7 | DMN | R | 289 | DefaultB_PFCv_3 | IFG | - | 1.18 |
| 8 | SN | R | 224 | SalVentAttnA_FrMed_2 | JPL | - | 1.15 |
| 9 | SN | L | 70 | SalVentAttnA_ParMed_1 | PCu/PCgG | - | 1.14 |
| 10 | FPN | L | 110 | ContC_Cingp_1 | PCgG | - | 1.13 |
| 11 | SN | R | 221 | SalVentAttnA_ParMed_1 | pCgG/aCgG | - | 1.11 |
| 12 | FPN | R | 247 | ContA_IPS_2 | SMG | - | 1.08 |
| 13 | SN | R | 222 | SalVentAttnA_ParMed_2 | PCu/PCgG | - | 1.08 |
| 14 | SN | R | 233 | SalVentAttnB_PFCmp_2 | SFG | - | 1.07 |
| 15 | FPN | L | 91 | ContA_IPS_2 | L-SPL/SMG | - | 1.07 |

|  | Alcohol Seeking/Sex Component | | | | | | |
| --- | --- | --- | --- | --- | --- | --- | --- |
|  | Network | Hemisphere | Schaefer 300 index | Schaefer 300 region | Description | Sign | Strength |
| 1 | DMN | R | 277 | DefaultA_pCunPCC_3 | PCu | + | 1.54 |
| 2 | SN | L | 67 | SalVentAttnA_Ins_2 | FO | + | 1.16 |
| 3 | SN | R | 216 | SalVentAttnA_Ins_1 | aINS | + | 1.13 |
| 4 | SN | L | 65 | SalVentAttnA_ParOper_2 | PO/SMG | + | 0.91 |
| 5 | SN | R | 220 | SalVentAttnA_FrMed_1 | R-ACgG | + | 0.85 |
| 6 | SN | L | 78 | SalVentAttnB_Ins_2 | FO/Ins | + | 0.81 |
| 7 | DMN | L | 126 | DefaultB_IPL_1 | AG | + | 0.74 |
| 8 | FPN | R | 251 | ContA_PFCl_2 | IFG/MFG | + | 0.71 |
| 9 | DMN | L | 114 | DefaultA_pCunPCC_1 | PCu | + | 0.71 |
| 10 | SN | R | 224 | SalVentAttnA_FrMed_2 | JPL | + | 0.70 |
| 11 | SN | L | 76 | SalVentAttnB_PFCl_2 | MFG | + | 0.68 |
| 12 | SN | L | 69 | SalVentAttnA_FrOper_1 | PCu/PCgG | - | 0.99 |
| 13 | SN | L | 74 | SalVentAttnB_IPL_1 | SMG | - | 0.79 |
| 14 | SN | L | 64 | SalVentAttnA_ParOper_1 | SMG | - | 0.68 |
| 15 | DMN | R | 282 | DefaultA_PFCm_5 | ACgG | - | 0.66 |

|  | Education Component | | | | | | |
| --- | --- | --- | --- | --- | --- | --- | --- |
|  | Network | Hemisphere | Schaefer 300 index | Schaefer 300 region | Description | Sign | Strength |
| 1 | SN | R | 221 | SalVentAttnA_ParMed_1 | pCgG/aCgG | + | 1.22 |
| 2 | DMN | R | 289 | DefaultB_PFCv_3 | IFG | + | 1.12 |
| 3 | DMN | L | 141 | DefaultC_Rsp_1 | pCgG | + | 1.01 |
| 4 | DMN | L | 112 | DefaultA_PFCd_1 | SFG | + | 1.00 |
| 5 | DMN | L | 132 | DefaultB_PFCd_5 | SFG | + | 0.92 |
| 6 | SN | R | 222 | SalVentAttnA_ParMed_2 | PCu/PCgG | + | 0.92 |
| 7 | DMN | R | 274 | DefaultA_PFCd_1 | MFG | + | 0.91 |
| 8 | SN | R | 226 | SalVentAttnB_IPL_1 | FP | - | 1.45 |
| 9 | SN | R | 219 | SalVentAttnA_FrOper_1 | CO | - | 1.41 |
| 10 | SN | L | 73 | SalVentAttnA_FrMed_2 | SFG | - | 1.31 |
| 11 | SN | R | 216 | SalVentAttnA_Ins_1 | aINS | - | 1.17 |
| 12 | SN | R | 218 | SalVentAttnA_Ins_3 | CO/mINS | - | 0.91 |
| 13 | SN | R | 221 | SalVentAttnA_ParMed_1 | pCgG/aCgG | - | 0.91 |
| 14 | SN | R | 217 | SalVentAttnA_Ins_2 | pINS | - | 0.90 |
| 15 | SN | R | 224 | SalVentAttnA_FrMed_2 | JPL | - | 0.84 |

**Table S1.** Top brain regions per component (top 5%, 15 regions). Index refers to Schaefer 300 parcellation (Schaefer et al., 2018)⁠ ACgG: Anterior Cingulate Gyrus, ACgG/JPL: Anterior Cingulate Gyrus/Juxtapositional Lobule, aINS: Ventral Anterior Insula, aINS/OFC: Anterior Insula/Orbitofrontal Cortex, CO/mINS: Central Operculum/Middle Insular Cortex, FO: Frontal Operculum, FO/OFC: Frontal Operculum/Lateral Orbitofrontal Cortex, FrP: Frontal Pole, IFG: Inferior Frontal Gyrus, IFG/MFG: Inferior/Middle Frontal Gyrus, INS: Insula, JPL: Juxtapositional Lobule Cortex, LOC: Lateral Occipital Cortex (superior), MFG: Middle Frontal Gyrus, MFG/IFG: Middle/Inferior Frontal Gyrus, MTG: Middle Temporal Gyrus, PCgG: Posterior Cingulate Cortex (retrosplenial), pCgG/aCgG: Cingulate Gyrus (posterior and anterior), PCu: Precuneus (anterior/dorsal), PCu/PCgG: Precuneus/Posterior Cingulate Gyrus, pINS: Ventral Posterior Insula, PO: Parietal Operculum, PoG/PrG: Postcentral/Precentral Gyrus (medial), extends into posterior Cingulate Gyrus (anteriorly) and Precuenus (posteriorly), PrG: Precentral Gyrus, PrG/IFG: Precentral/Inferior Frontal Gyrus, SFG: Superior Frontal Gyrus/Paracingulate Gyrus, SFG/MFG: Superior/Middle Frontal Gyrus, SMG: Supramarginal Gyrus (anterior), SPL/SMG: Superior Parietal Lobule/Supramarginal Gyrus (posterior)
